# Supplementary material for: A Benchmark of methods for SARS-CoV-2 whole genome sequencing and development of a more sensitive method
Source: Front Genet. 2025 Aug 15;16:1516791. doi: 10.3389/fgene.2025.1516791 (PMC12394145; doi:10.3389/fgene.2025.1516791)
Supplement: Supplementary file 3 [file Supplementaryfile2.docx]

**Supplementary figures**


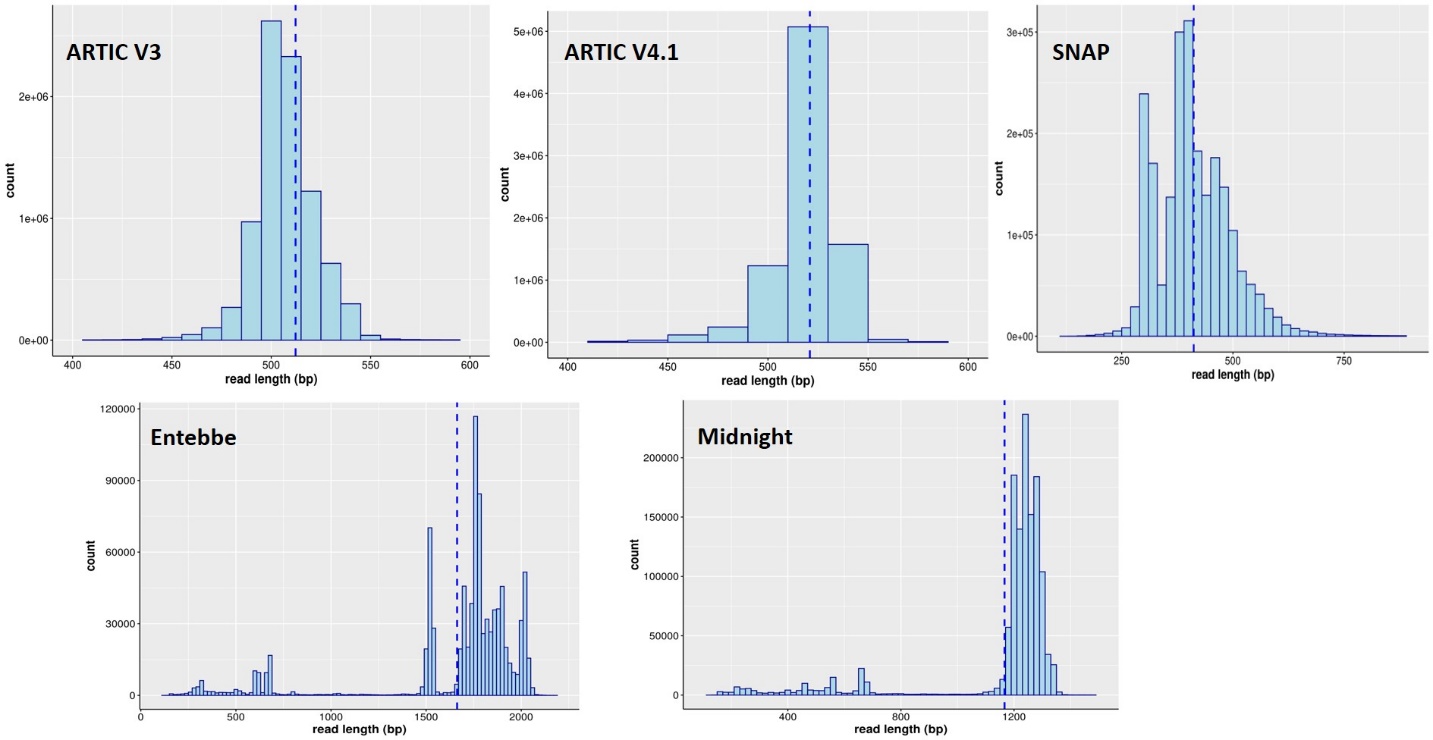


Supplementary Figure S1: Raw read length profiles. SARS-CoV-2 samples were processed following five different protocols: ARTIC v3, ARTIC v4.1, SNAP, Entebbe, and Midgnight to generated sequencing libraries. Libraries were sequenced on the Oxford Nanopore Technologies PromethION. The profile of raw reads generated is shown here. Blue dotted line shows the median read length.


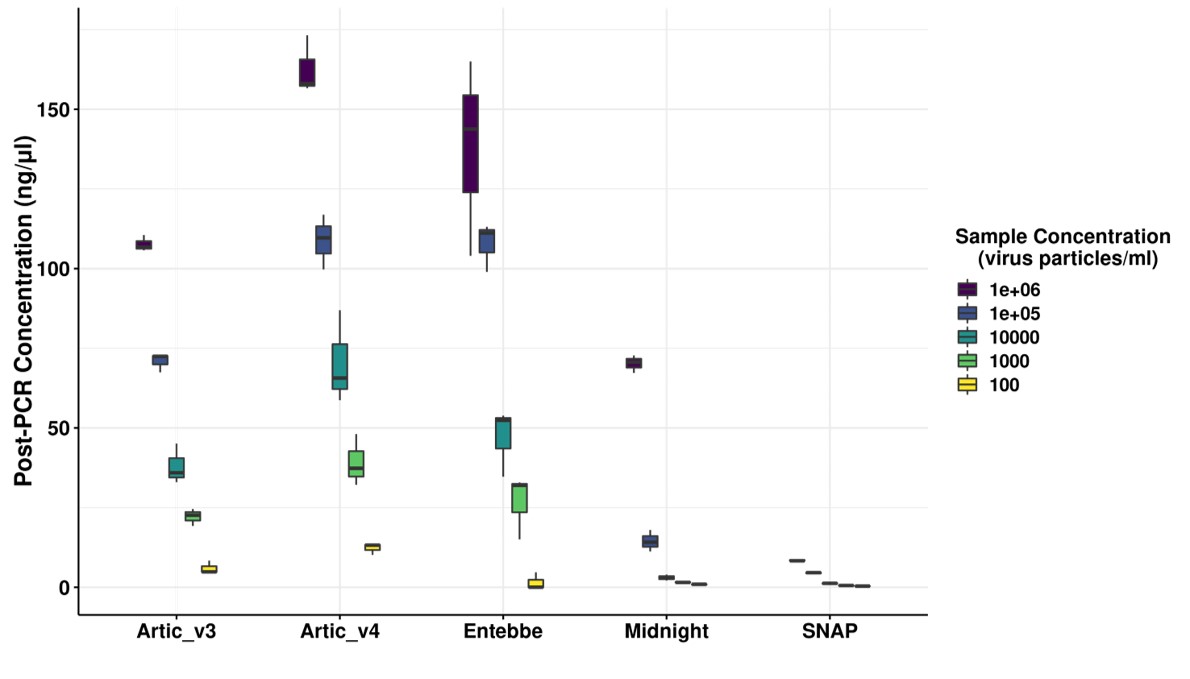


Supplementary Figure S2: Post-PCR concentration of synthetic genome amplicons. The Twist Biosciences synthetic genome representing wildtype SARS-CoV-2 was serial diluted and the samples processed in triplicate following five different protocols: ARTIC v3, ARTIC v4, Entebbe, Midnight, and SNAP. The concentration of purified PCR amplicons was measured and shown here.


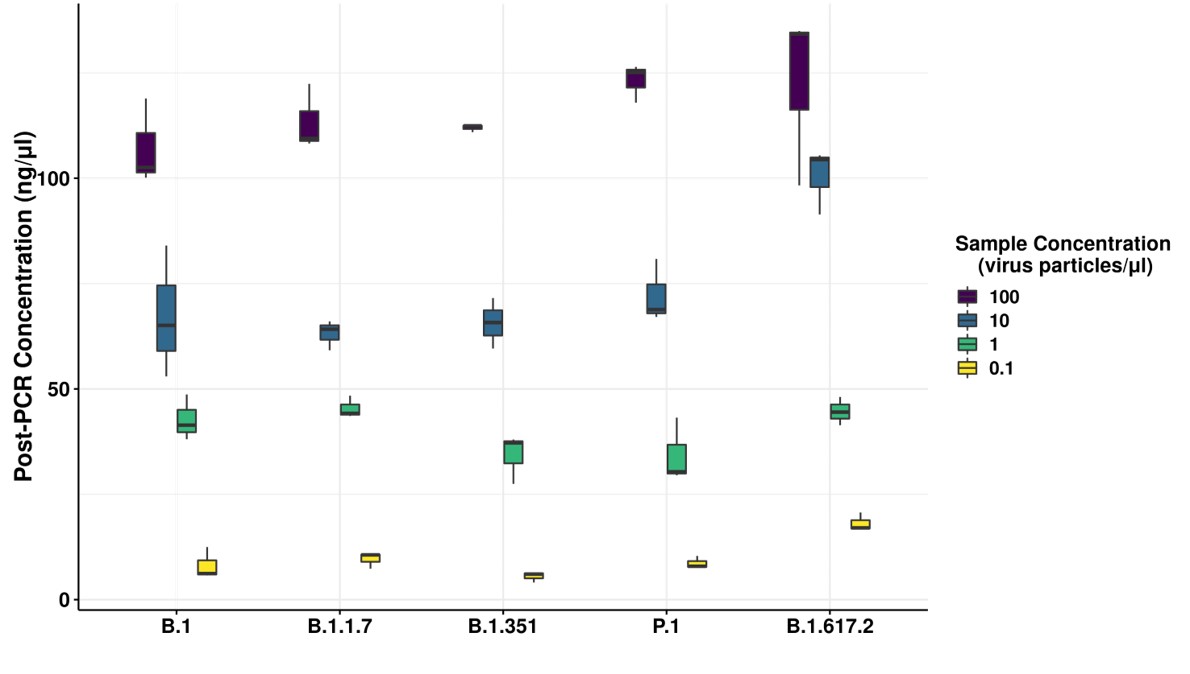


Supplementary Figure S3: Concentration of PCR amplicons of SARS-CoV-2 cell culture variants. The concentration of wildtype and 4 different SARS-CoV-2 variant samples was determined by digital PCR followed by normalisation of the samples. The normalised samples were serial diluted and sequencing libraries prepared following ARTIC v4.1 protocol. The concentration of purified PCR products is shown.


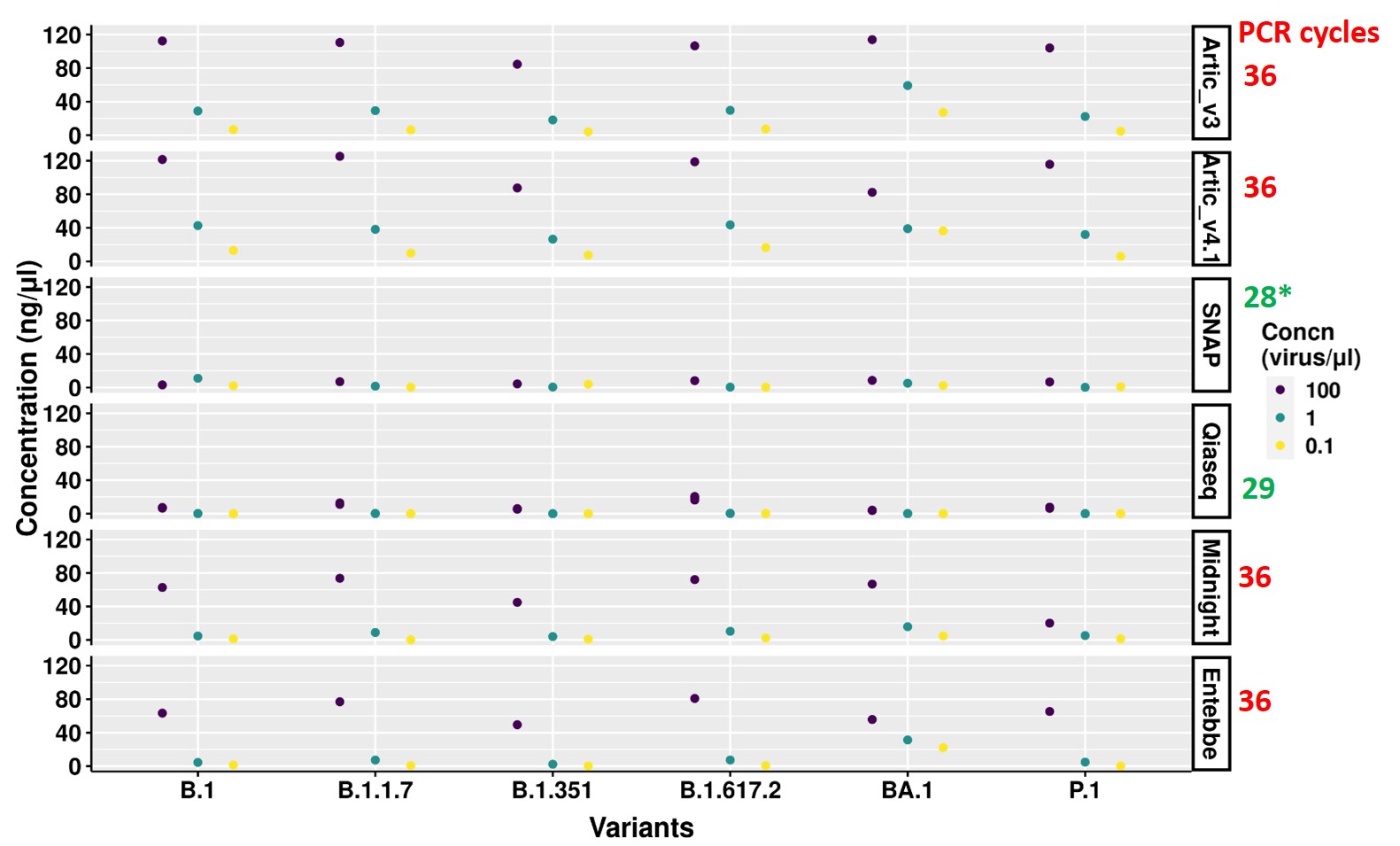


Supplementary Figure S4: Concentration of PCR amplicons of SARS-CoV-2 cell culture variants across protocols. Normalised and serial diluted wildtype and five different SARS-CoV-2 variant samples at 3 viral titres: 100, 1, and 0.1 particles/µL were processed following six different protocols: ARTIC v3, ARTIC v4.1, Qiaseq, SNAP, Entebbe, and Midnight. The concentration of purified PCR products is shown here. The number of PCR cycles performed for each protocol is shown in red on the right of the figure. For the SNAP protocol, we performed 36 cycles for BA.1 and 28 cycles for all other samples. For Qiaseq protocol, all samples were run in triplicate and three dots representing the three samples are shown albeit that they overlap.


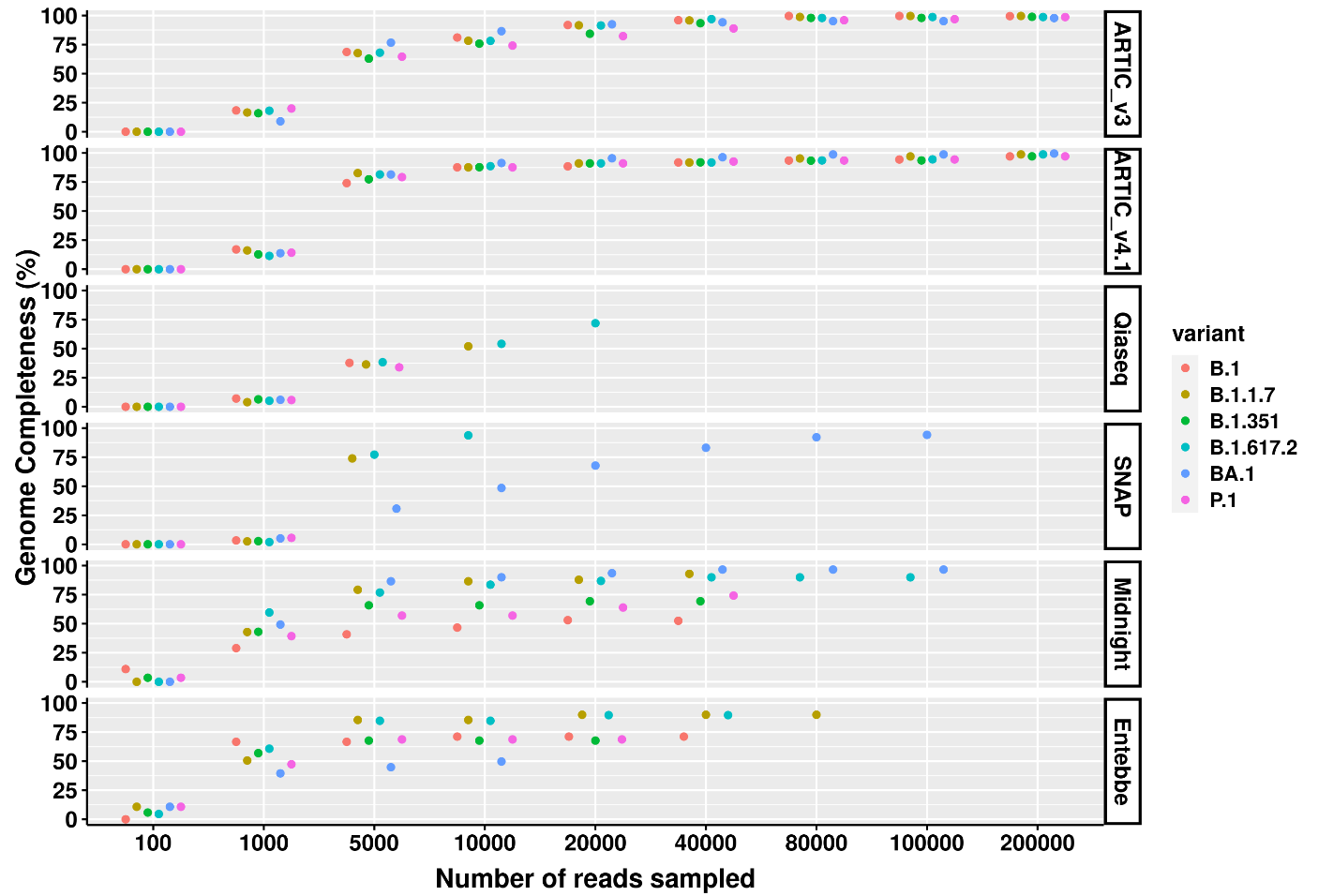


Supplementary Figure S5: SARS-CoV-2 genome completeness comparison across medium viral titre variants and protocols. Wildtype SARS-CoV-2 and five cell culture variant samples were processed for sequencing following six different protocols; ARTIC v3, ARTIC v4.1, Qiaseq, SNAP, Midnight, and Entebbe. Prepared cDNA libraries were sequenced on the PromethION and the data analysed using ARTIC pipeline to reconstruct the genomes using a set of randomly sub-sampled reads. At each set of sampled reads the coverage of the reconstructed genome was computed as the percentage of the fully reconstructed genome (without gaps represented by ‘N’s). Samples used were the normalised and serial dilutions at 1 viral particles/µL.


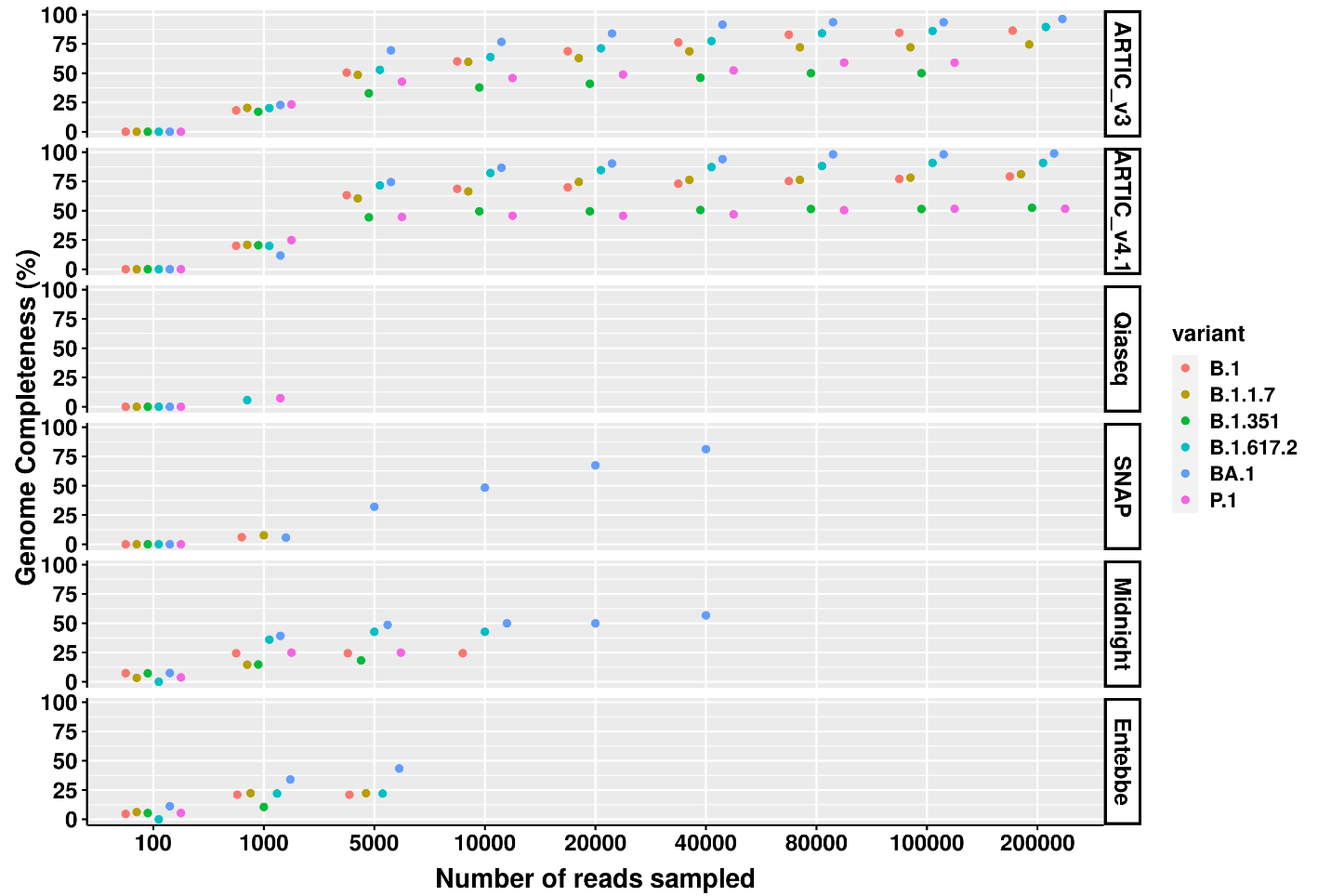


Supplementary Figure S6: SARS-CoV-2 genome completeness comparison across low viral titre variants and protocols. Wildtype SARS-CoV-2 and five cell culture variant samples were processed for sequencing following six different protocols; ARTIC v3, ARTIC v4.1, Qiaseq, SNAP, Midnight, and Entebbe. Prepared cDNA libraries were sequenced on the PromethION and the data analysed using ARTIC pipeline to reconstruct the genomes using a set of randomly sub-sampled reads. At each set of sampled reads the coverage of the reconstructed genome was computed as the percentage of the fully reconstructed genome (without gaps represented by ‘N’s). Samples used were the normalised and serial dilutions at 0.1 viral particles/µL.


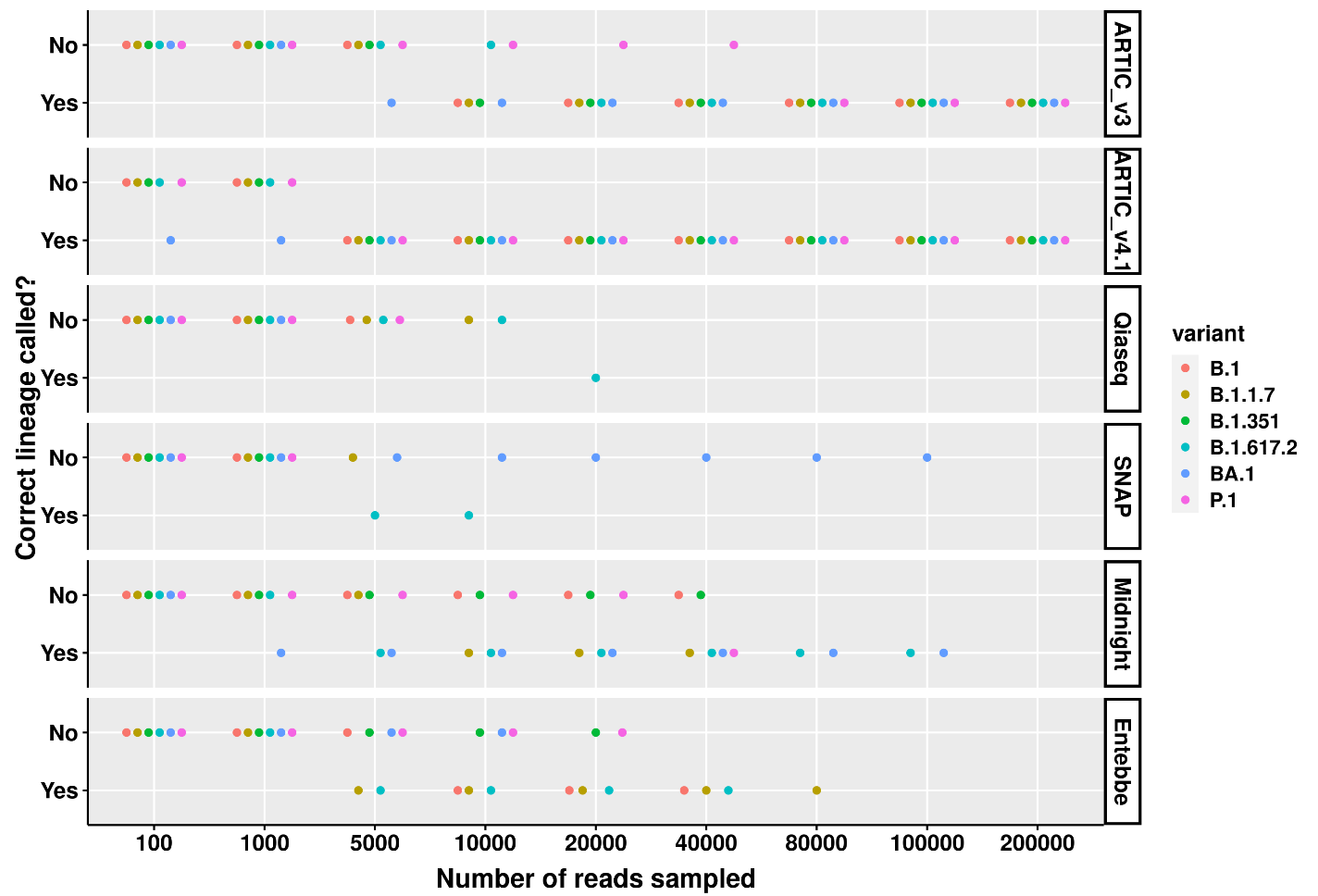


Supplementary Figure S7: Correct SARS-CoV-2 lineage calling comparison across medium viral titre variants and protocols. Wildtype SARS-CoV-2 and five cell culture variant samples were processed for sequencing following six different protocols; ARTIC v3, ARTIC v4, Qiaseq, SNAP, Midnight, and Entebbe. Prepared cDNA libraries were sequenced on the PromethION and the data analysed using ARTIC pipeline to reconstruct the genomes using a set of randomly sub-sampled reads. At each set of sampled reads the Pangolin pipeline (O'Toole et al., 2021) was used to assign Pango lineages (Rambaut et al., 2020) to the reconstructed genome. The figure shows whether the reconstructed genome allowed for the correct lineage to be assigned or not. Samples used were the normalised and serial dilutions at 1 viral particles/µL.


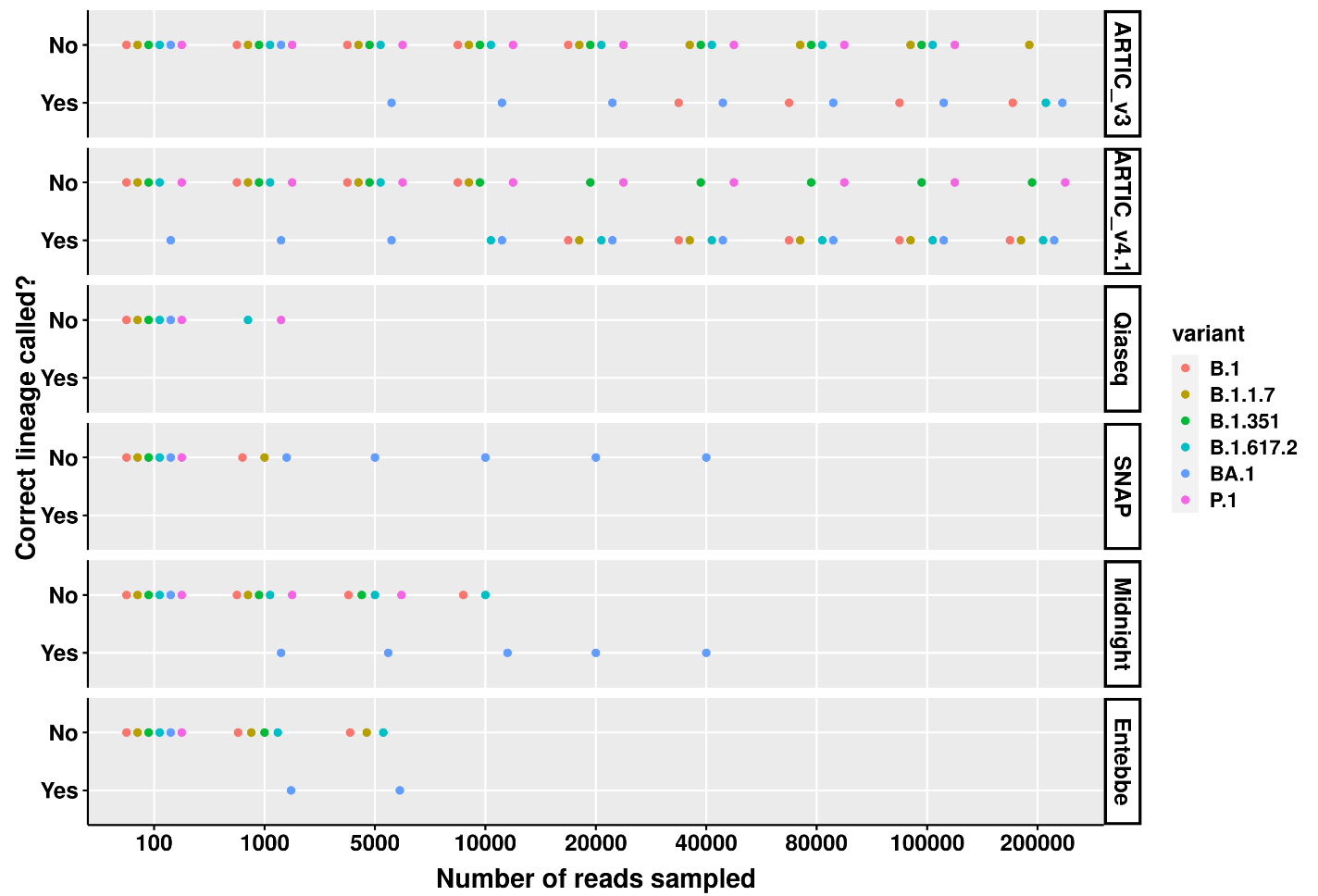


Supplementary Figure S8: Correct SARS-CoV-2 lineage calling comparison across low viral titre variants and protocols. Wildtype SARS-CoV-2 and five cell culture variant samples were processed for sequencing following six different protocols: ARTIC v3, ARTIC v4, Qiaseq, SNAP, Midnight, and Entebbe. Prepared cDNA libraries were sequenced on the PromethION and the data analysed using ARTIC pipeline to reconstruct the genomes using a set of randomly sub-sampled reads. At each set of sampled reads the Pangolin pipeline (O'Toole et al., 2021) was used to assign PANGO lineages (Rambaut et al., 2020) to the reconstructed genome. The figure shows whether the reconstructed genome allowed for the correct lineage to be assigned or not. Samples used were the normalised and serial dilutions at 0.1 viral particles/µL.


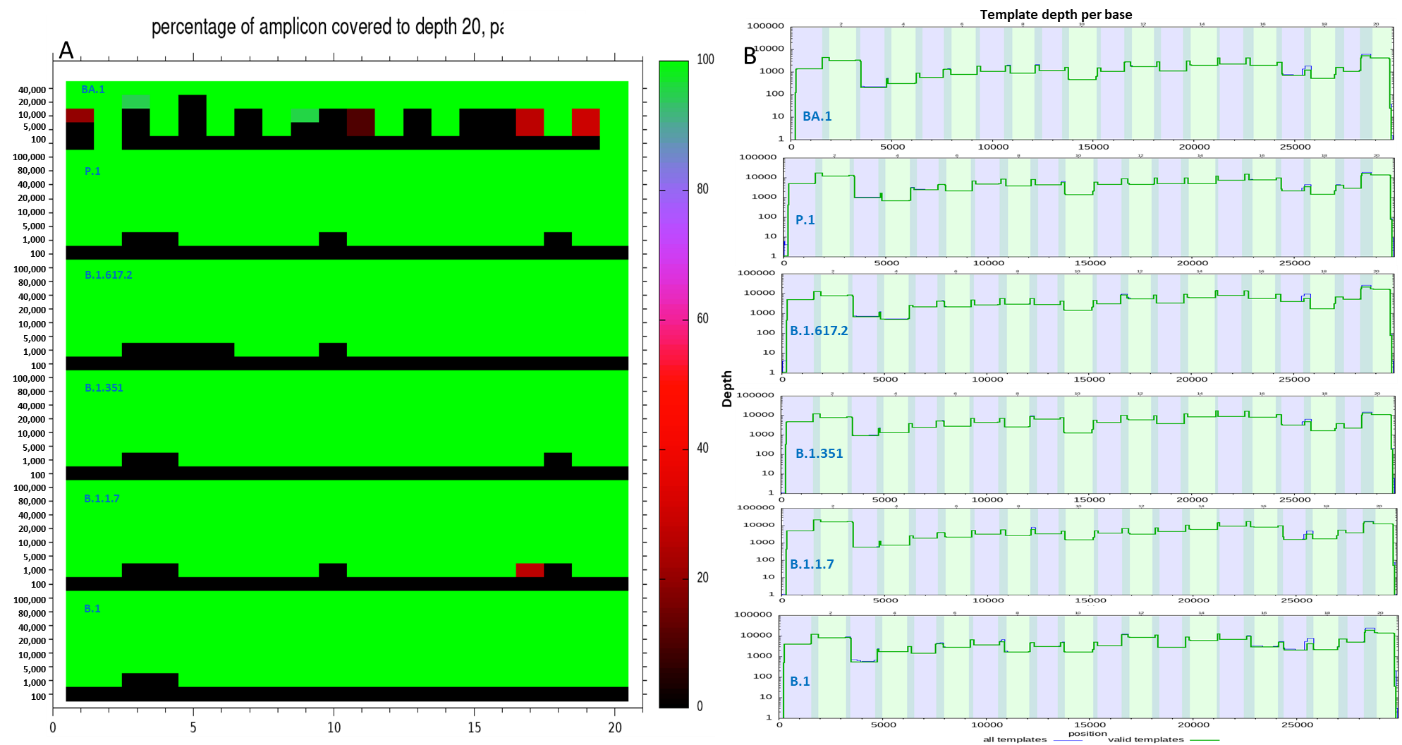


Supplementary Figure S9: Genome coverage for Entebbe protocol. A) Heatmap showing percentage of Entebbe protocol primers covered at ≥ 20X using the number of reads subsampled and across 6 SARS-CoV-2 lineages; B.1, B.1.1.7, B.1.351, B.1.617.2, P.1, and BA.1 at high viral titres. The primer numbers are shown on the X-axis while the subsampled reads are shown on the Y-axis. B) Genome coverage depth across SARS-CoV-2 lineages using all reads (no subsampling). The depth of coverage is shown on the y-axis. The bottom x-axis shows SARS-CoV-2 genomic position while top x-axis shows the primer number. The images were generated using the “plot-ampliconstats” function of Samtools (Li et al., 2009) and bam files containing aligned reads. The green lines indicate end-to-end primer-pair coverage while the blue lines indicate overall genomic region depth.


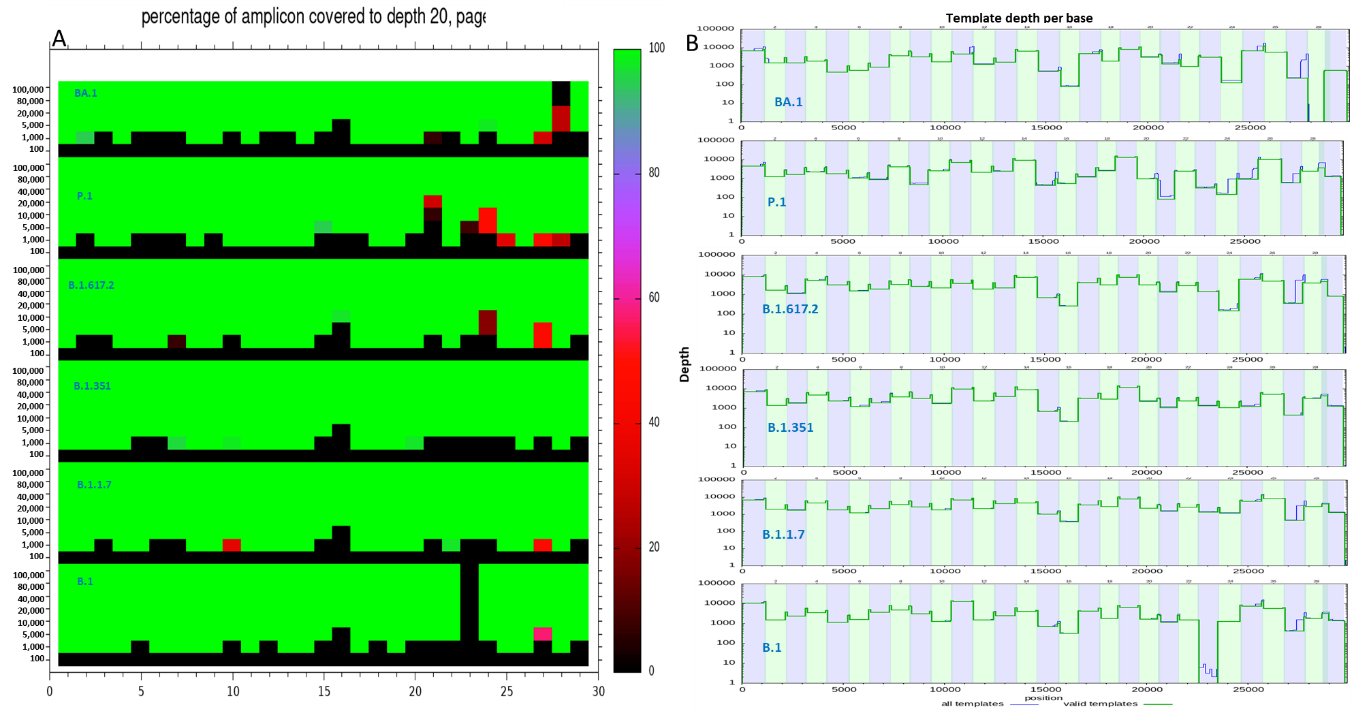


Supplementary Figure S10: Genome coverage for Midnight protocol. Figures A and B are same as Supplementary Figure S9 except the results are from Midnight protocol.


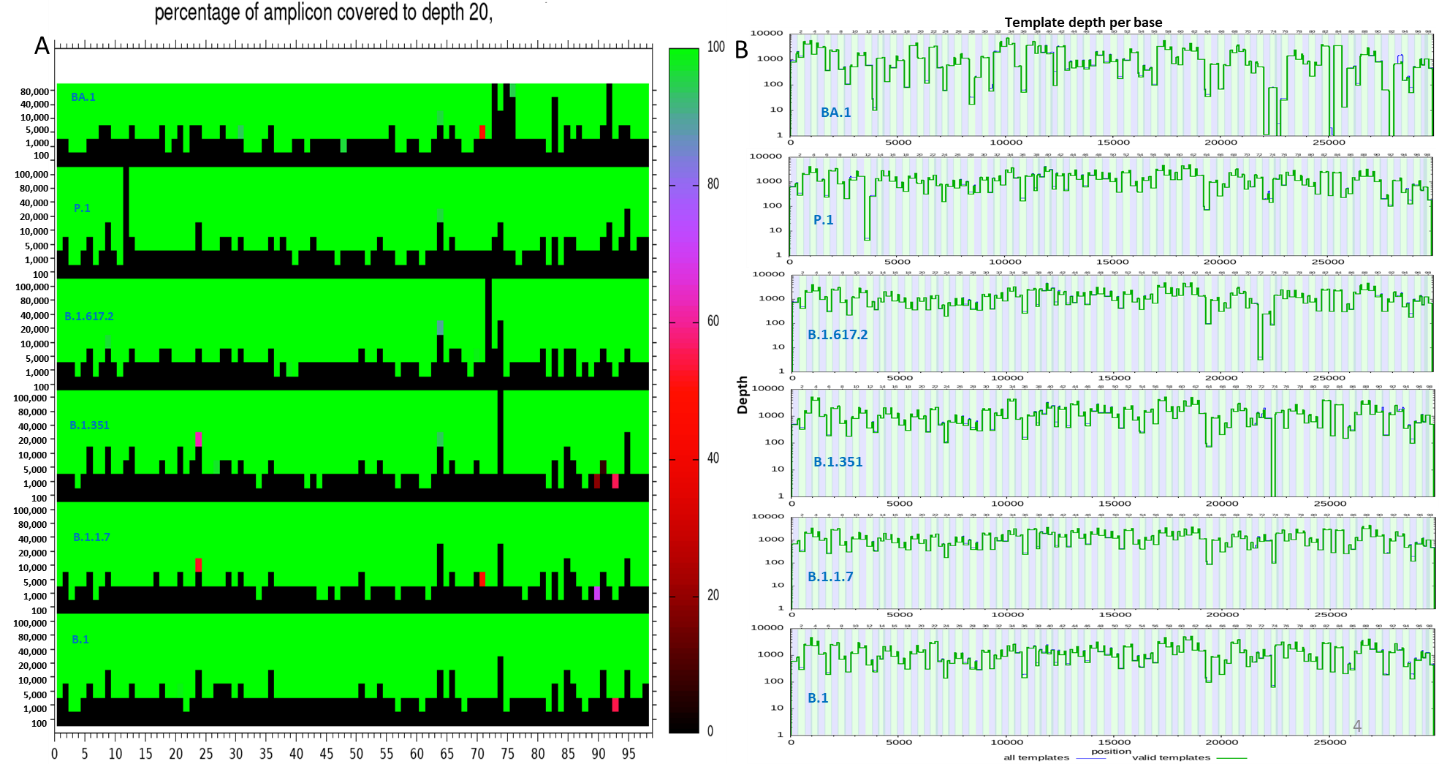


Supplementary Figure S11: Genome coverage for ARTIC v3 protocol. Figures A and B are same as Supplementary Figure S9 except the results are from Midnight protocol.


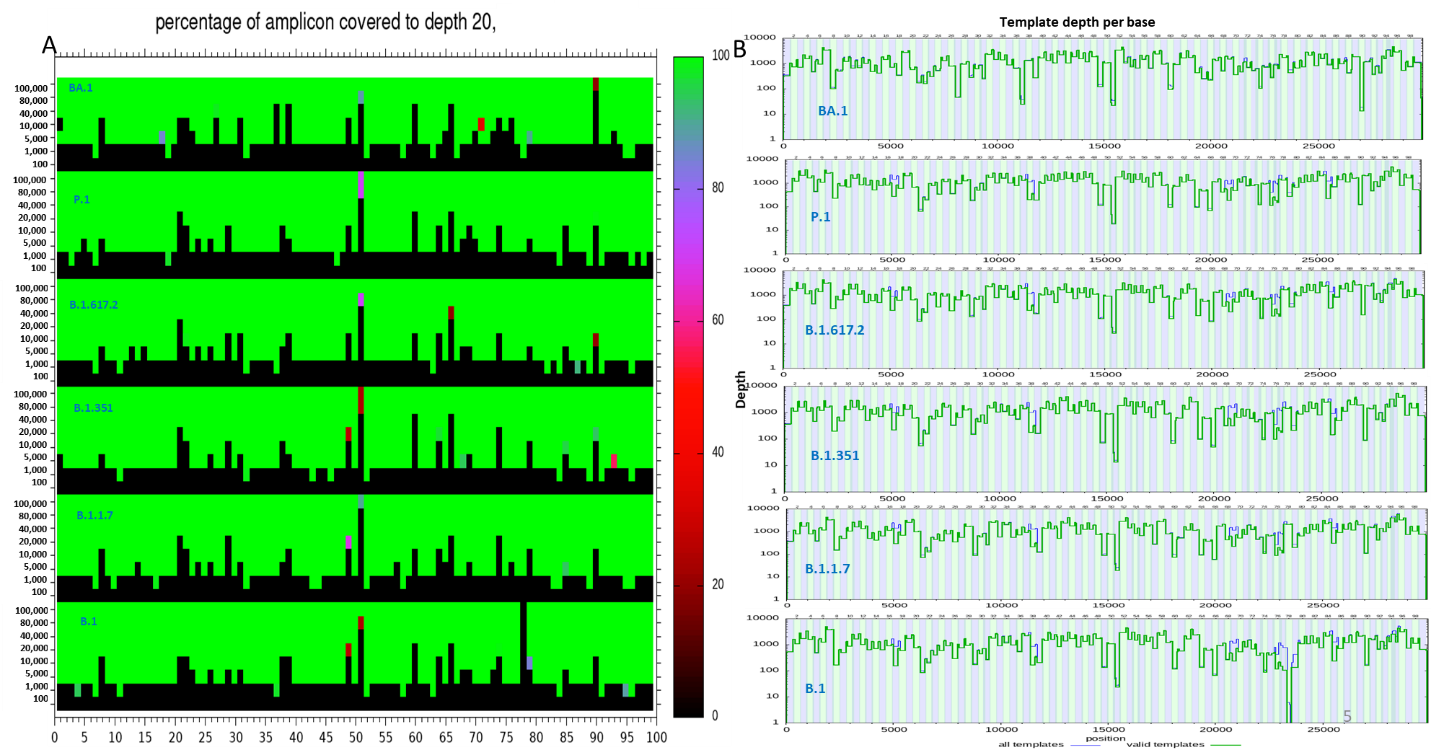


Supplementary Figure S12: Genome coverage for ARTIC v4.1 protocol. Figures A and B are same as Supplementary Figure S9 except the results are from Midnight protocol.


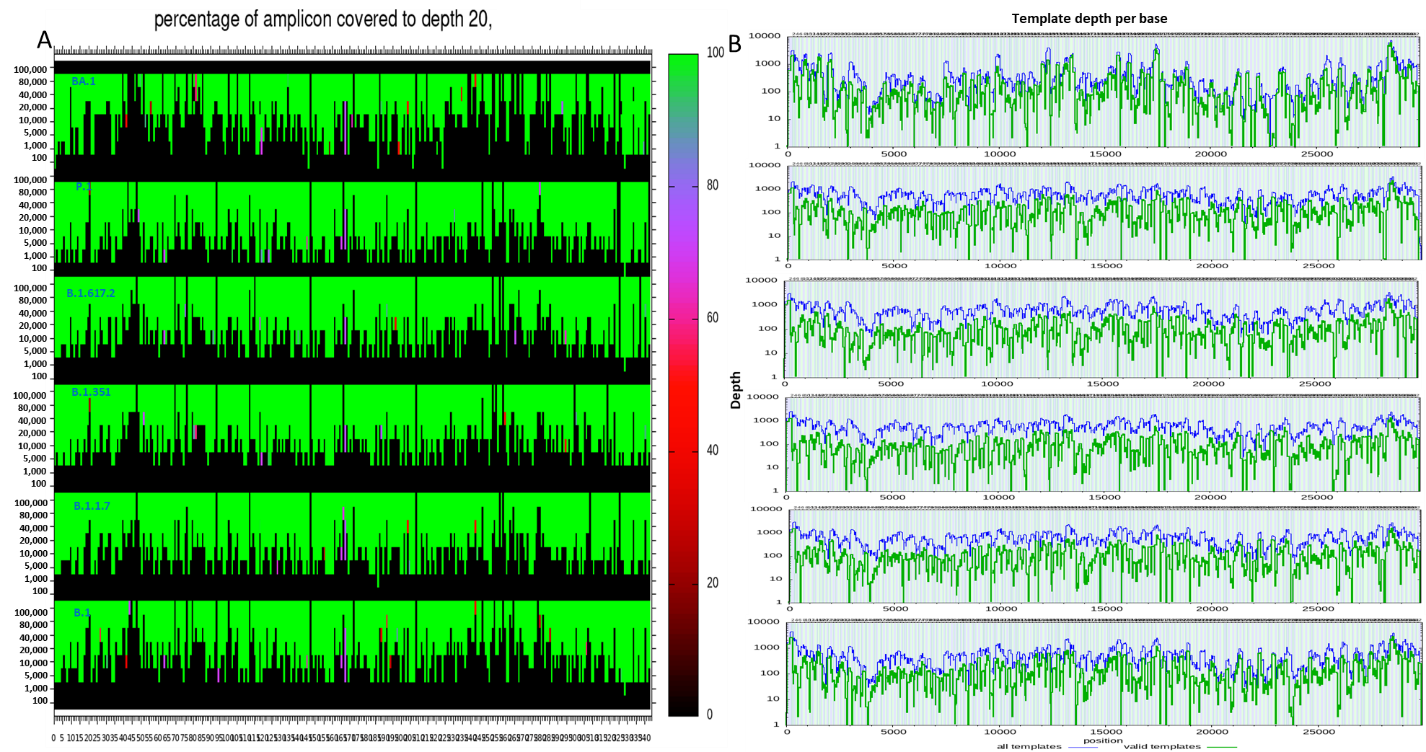


Supplementary Figure S13: Genome coverage for SNAP protocol. Figures A and B are same as Supplementary Figure S9 except the results are from Midnight protocol. The green lines indicate end-to-end primer-pair coverage while the blue lines indicate overall genomic region depth.


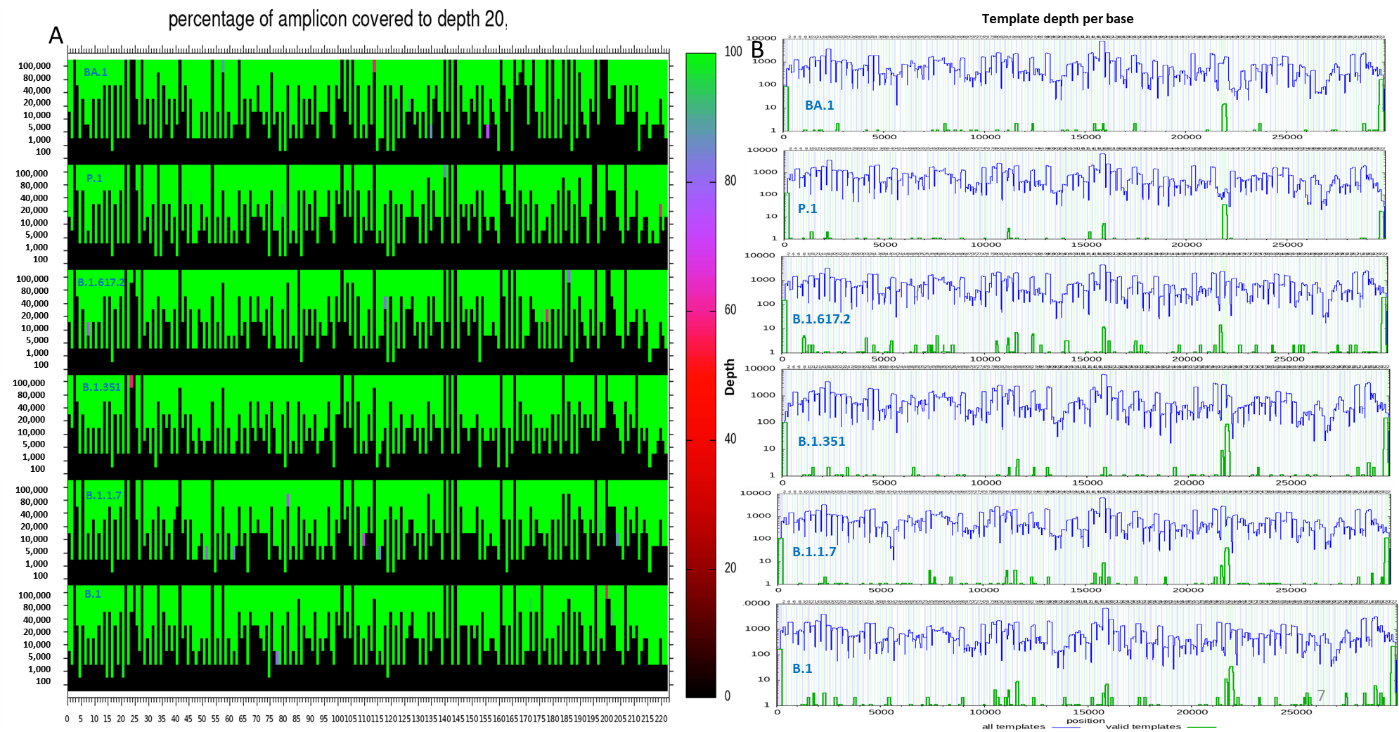


Supplementary Figure S14: Genome coverage for Qiaseq protocol. Figures A and B are same as Supplementary Figure S9 except the results are from Midnight protocol. The green lines indicate end-to-end primer-pair coverage while the blue lines indicate overall genomic region depth. The primer design did not allow for accurate calculation of primer coverage.


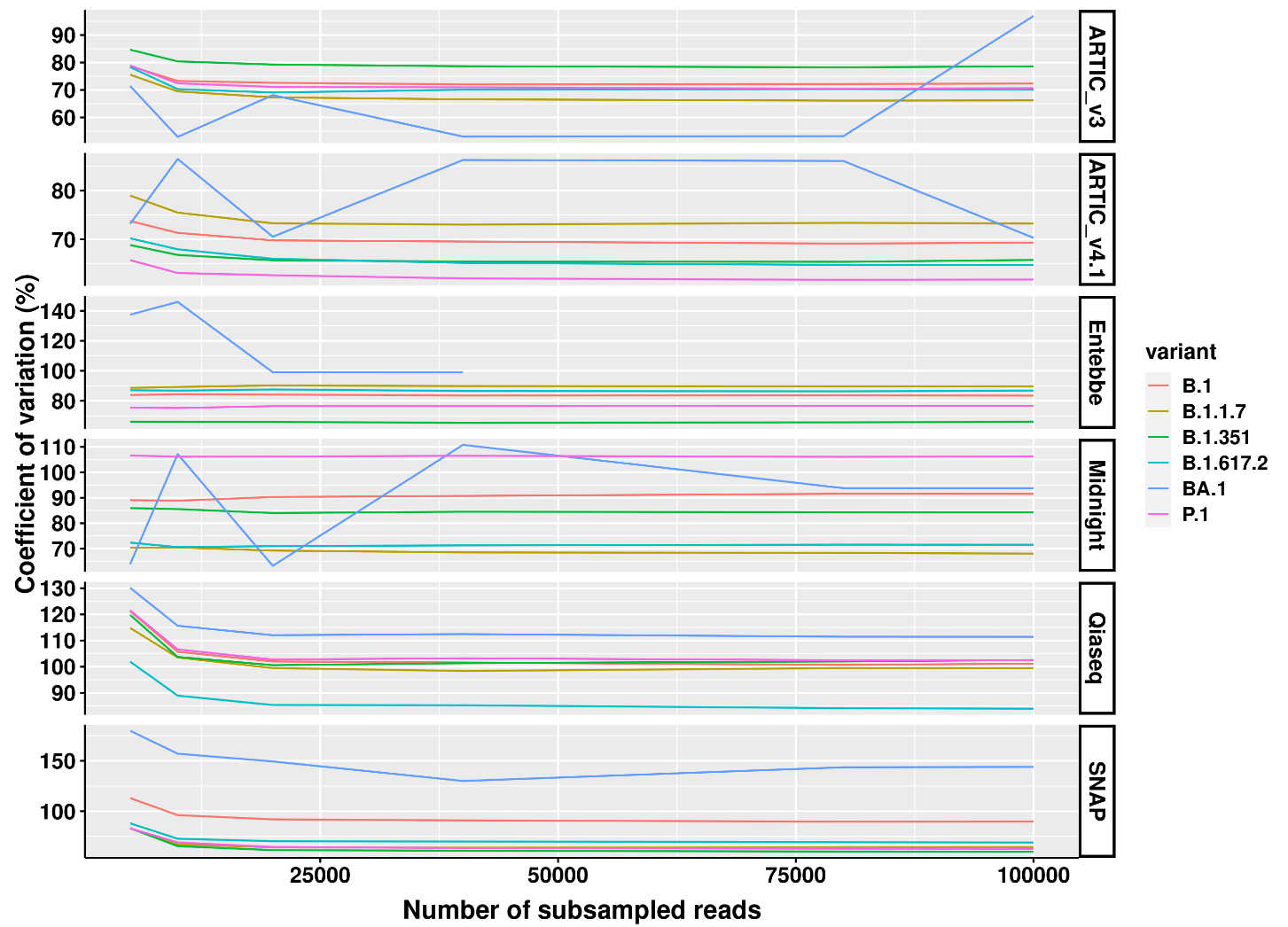


Supplementary Figure S15: Evenness of genome coverage. Six samples containing high viral titre SARS-CoV-2 lineages namely B.1, B.1.1.7, B.1.351, B.1.617.2, P.1, and BA.1 were processed with six protocols namely ARTIC v3, ARTIC v4.1, Entebbe, Midnight, Qiaseq, and SNAP. The reads generated were subsampled and aligned to the genome and the coverage determined using the “samtools depth” function of Samtools (Li et al., 2009). The coefficient of variation was determined as a percentage of the standard deviation of the coverage divided by the mean.


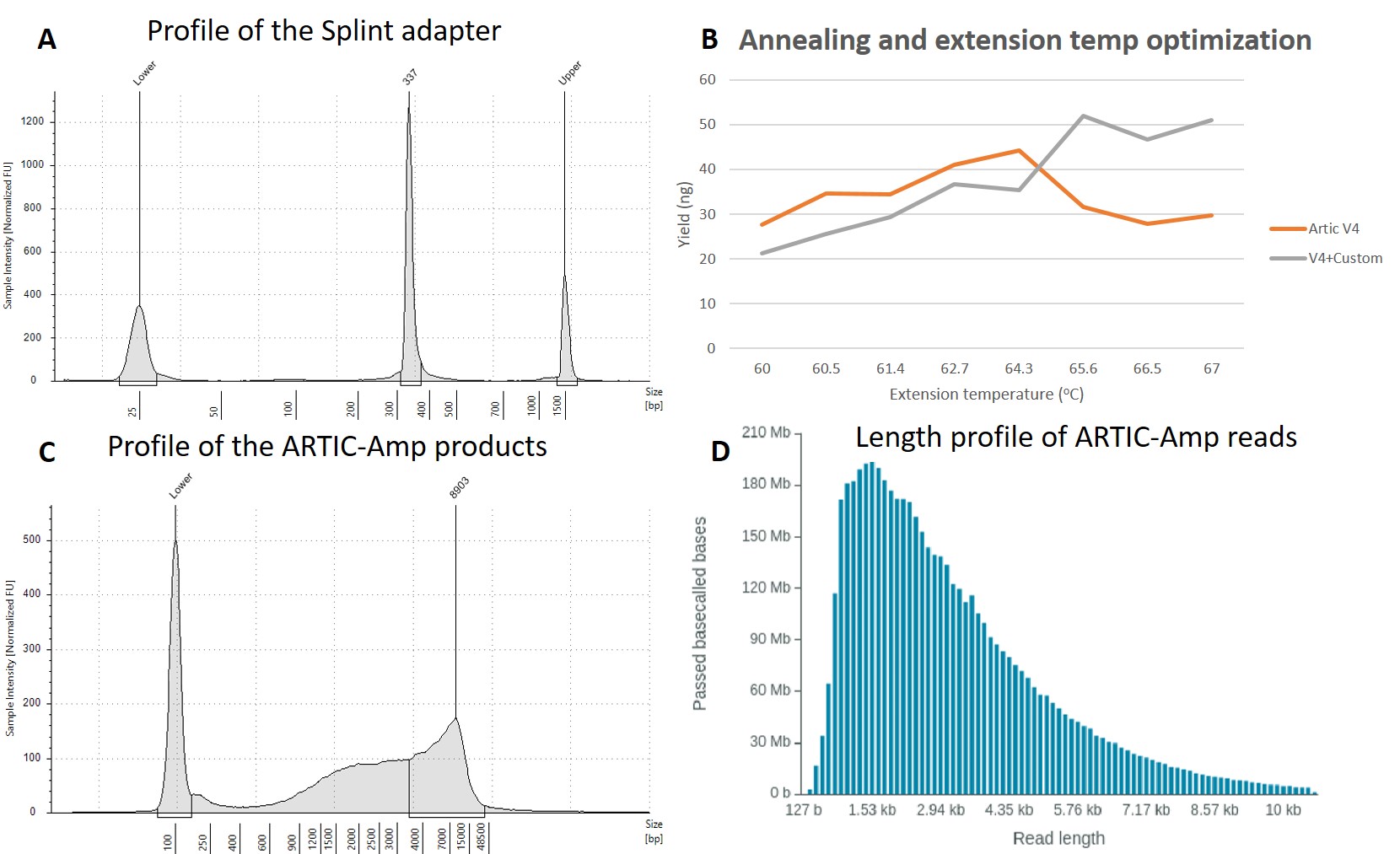


Supplementary Figure S16: ARTIC-Amp protocol product evaluation. A) Profile of the 330 bp ‘splint’ sequence amplified. The splint is used in circularisation of ARTIC amplicons via Gibson assembly. A Tapestation-generated image is shown here (). B) Optimisation of annealing and extension temperature for the ARTIC v4.1 primers that were modified to add a 29 bp tag sequence that would enable circularisation of molecules in conjunction with the ‘splint’ via Gibson assembly. C) Profile of ARTIC-Amp final products following circularisation of amplicons and rolling circle amplification. A peak length of 8.9 kb was achieved. A Tapestation-generated image is shown here () D) Profile of reads generated from sequencing of libraries prepared through the ARTIC-Amp protocol. The image is generated by MiniKNOW (Oxford Nanopore Technologies, UK).


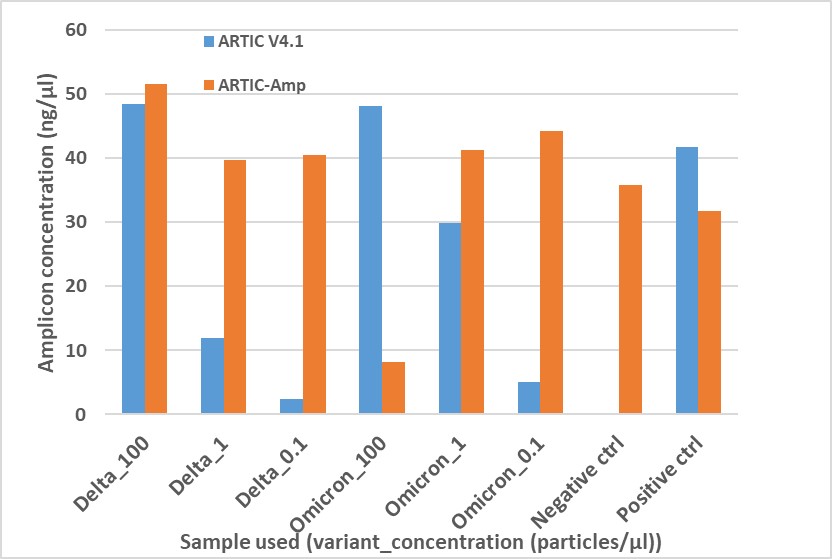


Supplementary Figure S17: Amplicon concentration comparison between ARTIC v4.1 and ARTIC-Amp protocols. Four primer sets targeting regions in four SARS-CoV-2 genes: N, ORF7a, ORF1a, and Spike, respectively were used to prepare sequencing libraries either following the ARTIC v4.1 protocol or our inhouse protocol termed ARTIC-Amp. The ARTIC-Amp protocol takes the final products of the ARTIC v4.1 protocol and circularises them via Gibson assembly followed by isothermal rolling circle amplification as described previously (Volden et al., 2018). Two SARS-CoV-2 variant namely B.1.617.2 (Delta) and Omicron (BA.1) samples were processed at three different concentrations: 100, 1, and 0.1 particles/µL. Low viral load samples (0.1 particles/µL) were processed in triplicate and the average is shown here.


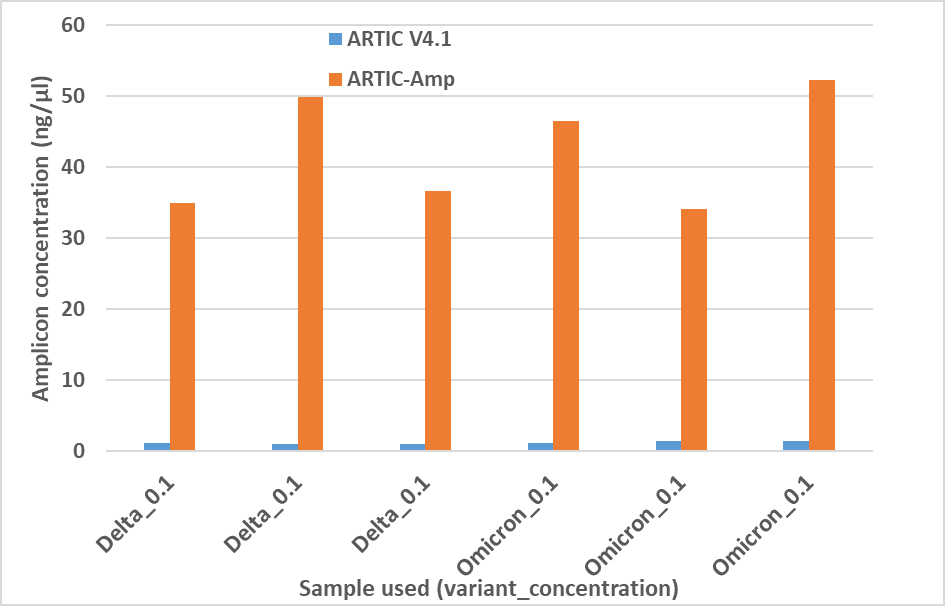


Supplementary Figure S18: Same as Supplementary Figure 17 but showing low viral load samples (0.1 particles/µL) which were processed in triplicate.


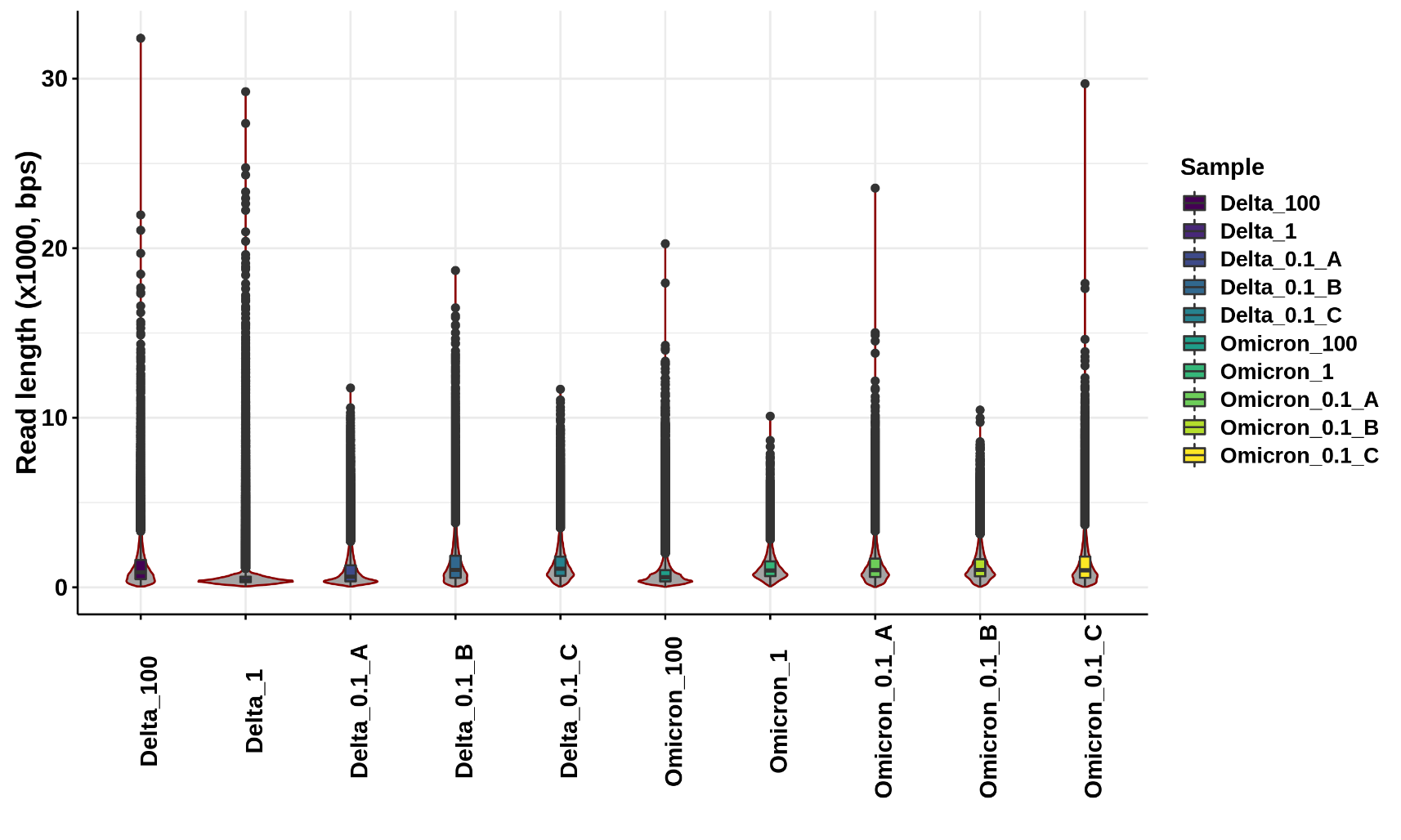


Supplementary Figure S19: Read length distribution of ARTIC-Amp libraries. Ten samples were processed following our inhouse ARTIC-Amp protocol and sequenced on the PromethION. The reads generated were trimmed of sequencing adapters and their lengths determined. The ten samples comprised two SARS-CoV-2 cell culture variants namely: B.1.617.2 (Delta) and BA.1 (Omicron). The samples were normalised and serial diluted to three concentrations, 100, 1, and 0.1 particles/µL. Low viral load samples (0.1 particles/µL) were processed in triplicate and shown here as A, B, and C.


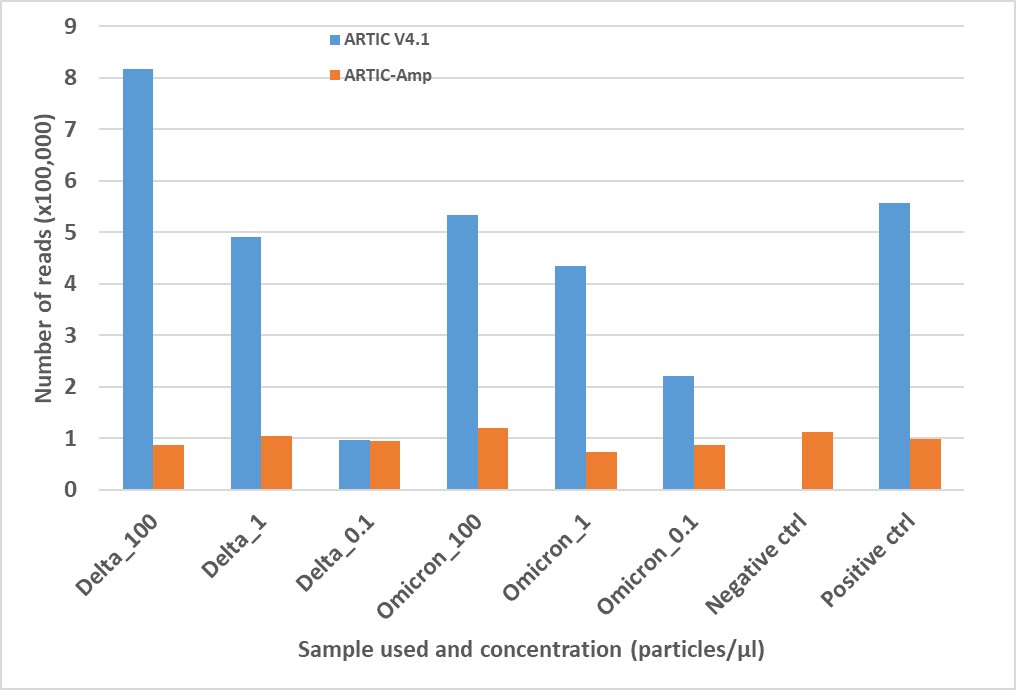


Supplementary Figure S20: Total number of reads generated from ARTIC v4.1 and ARTIC-Amp protocols. Same details as Supplementary Figure 17 but showing total number of reads generated following sequencing on the PromethION. Low viral load samples (0.1 particles/µL) were processed in triplicate and the average is shown here.


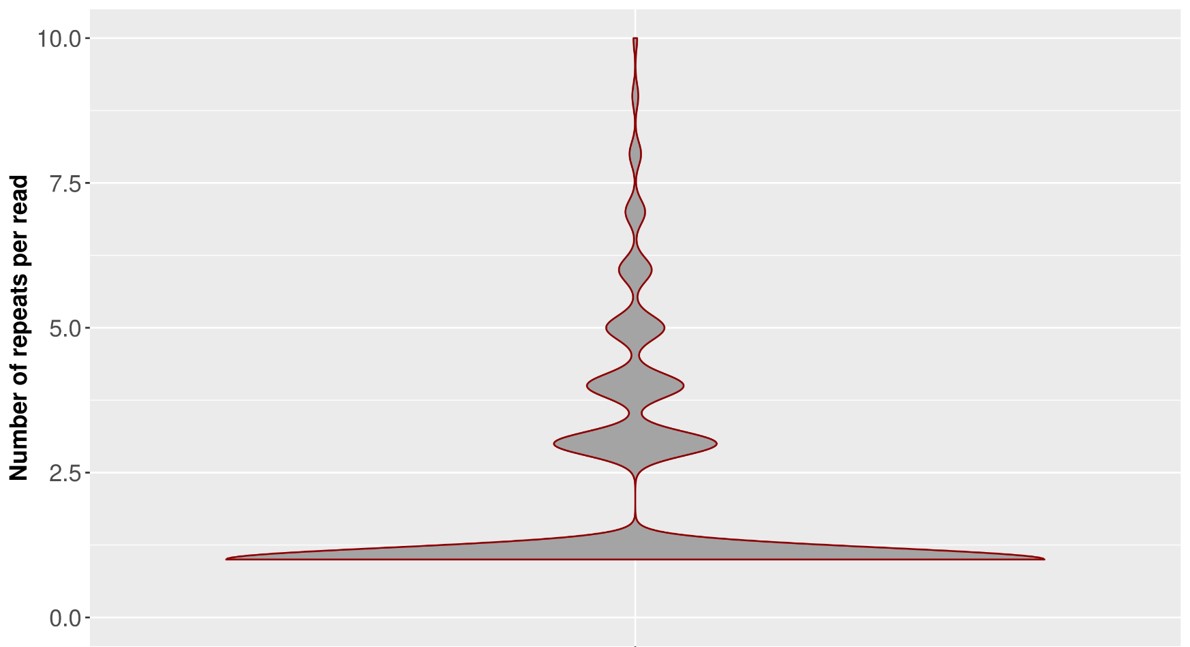


Supplementary Figure S21: Profile of number of repeats in reads generated through ARTIC-Amp protocol. Our inhouse ARTIC-Amp protocol includes rolling circle amplification of circularised molecules which creates repeats of the circularised molecules. We used R2C2 pipeline (Volden et al., 2018) to identify and count the number of repeats in each read.


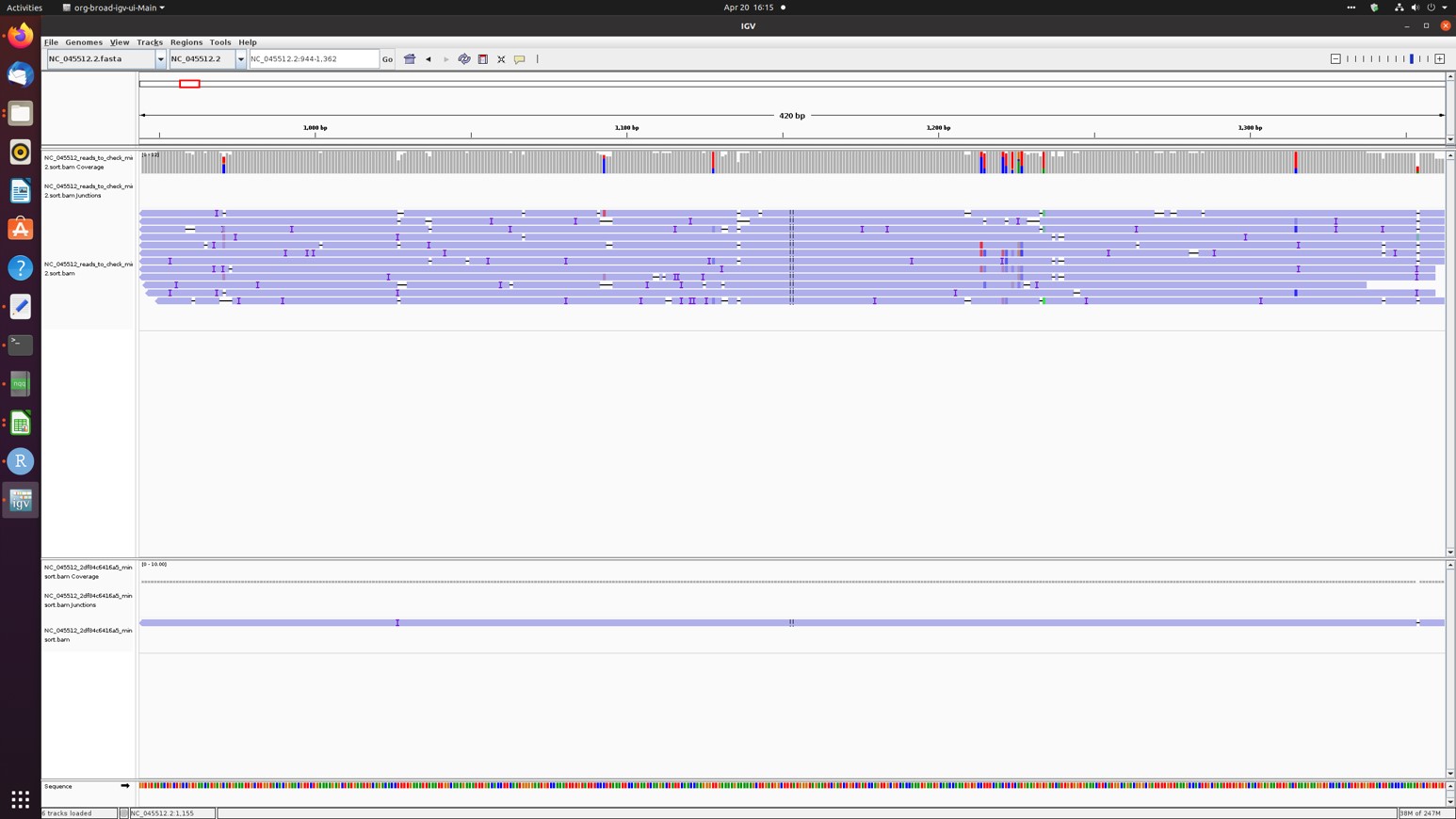


Supplementary Figure S22: Example of consensus error correction. IGV screen shot shows in the top panel a single read that had 13 repeats and the final consensus error-corrected final read in the bottom panel.


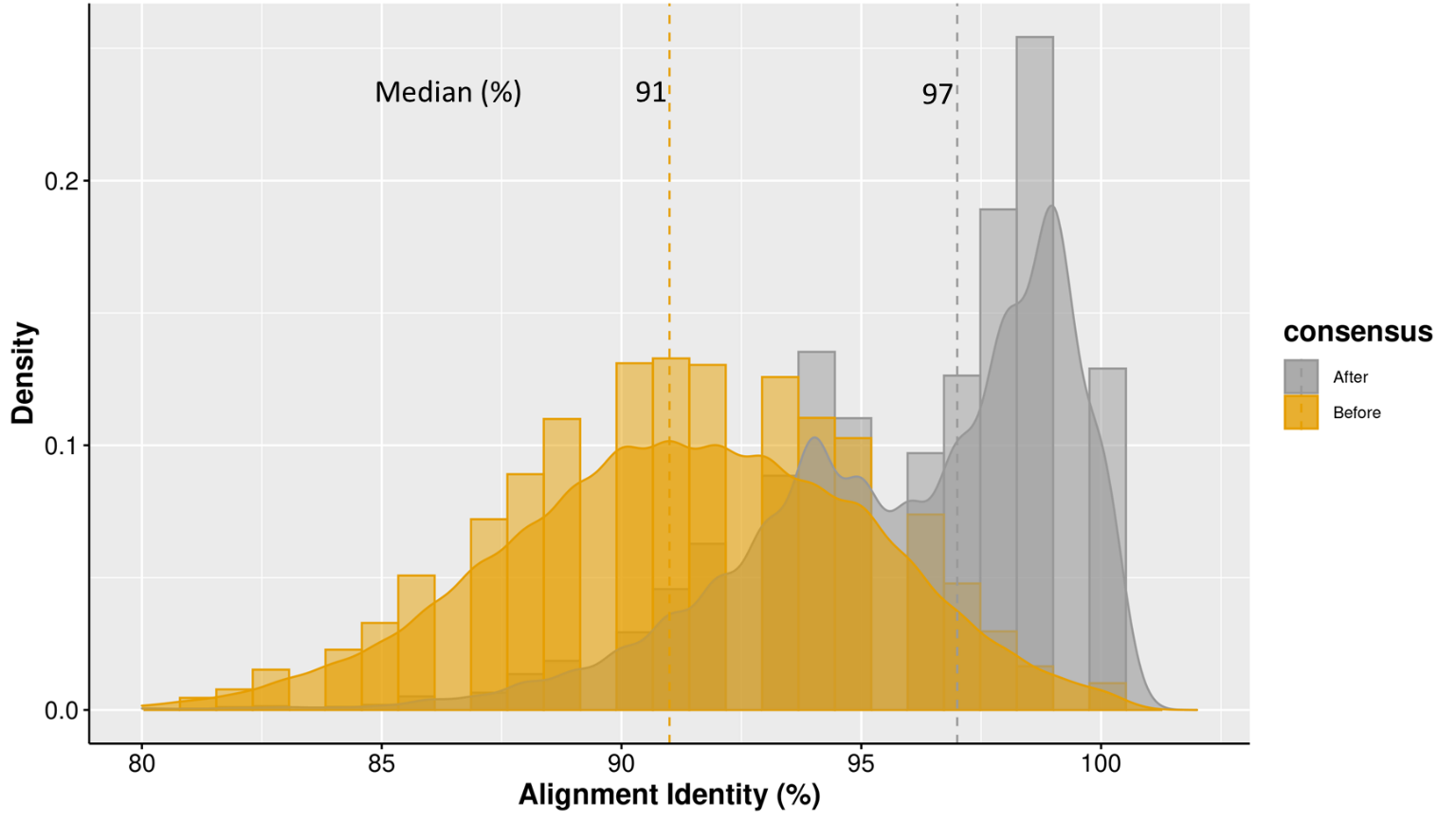


Supplementary Figure S23: Alignment identity improvement of reads generated by ARTIC-Amp protocol. Our in-house ARTIC-Amp protocol includes rolling circle amplification of circularised molecules which creates repeats of the circularised molecules. We used R2C2 pipeline (Volden et al., 2018) to identify repeats in each read and create a consensus error corrected final read for each molecule. The alignment identity of the reads before and after consensus error correction was determined and is shown here.


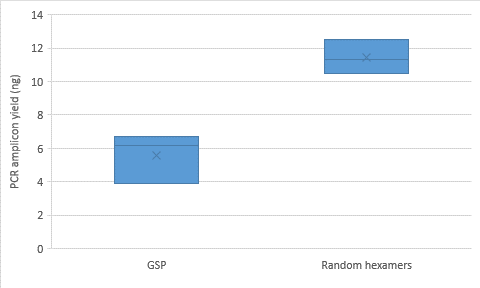


Supplementary Figure S24: PCR amplicon yield comparison. We compared the yield from PCR amplification of cDNA either prepared using gene-specific primers (GSP) or random hexamers during reverse transcription while performing Entebbe protocol. The Entebbe protocol uses GSP while ARTIC protocol uses random hexamers.


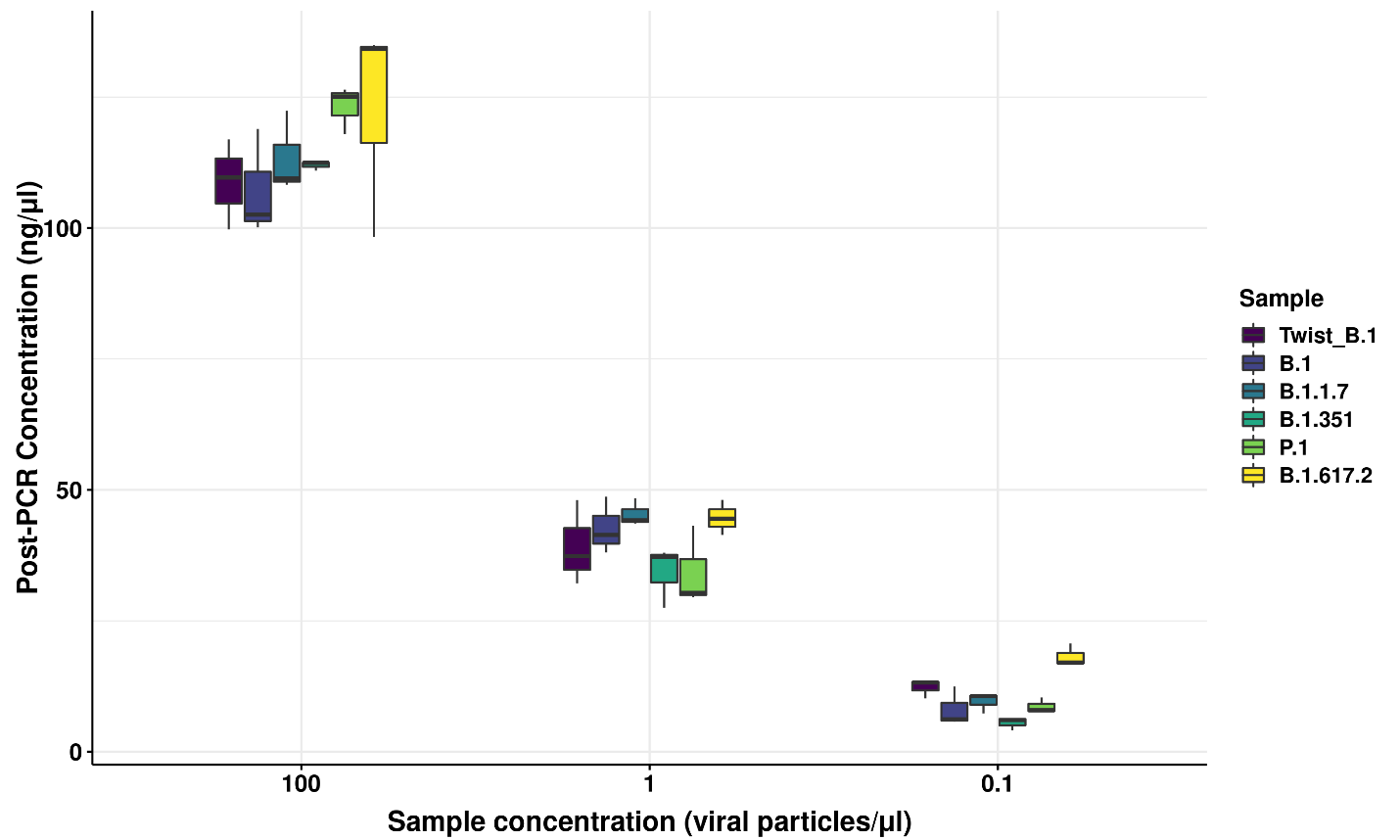


Supplementary Figure S25: Comparing the concentration of Twist synthetic control and cell culture variants normalised by ddPCR. The Twist Synthetic SARS-CoV-2 RNA Control 1 (here shown as Twist_B.1) whose commercial concentration is 1,000,000 particles per microliter was log serial diluted to obtain samples at 1x10^5^, 1x10^3^, and 1x10^2^ particles per millilitre. We processed these samples in triplicate following the ARTIC v4 protocol and determined the post-PCR amplicon concentrations which are shown here. Cell culture wildtype and variant SARS-CoV-2 virus were purified from cell culture supernatant and their concentrations determined by digital droplet PCR (ddPCR). The cell culture samples were normalised to 1000 particles/µL and then log serial-diluted to 100, 1, and 0.1 particles per microliter. The samples were processed following the ARTIC v4 protocol and the post-PCR amplicon concentrations determined which are shown here.
